# A Miniature Aerosol Sensor for Detecting Polydisperse Airborne Ultrafine Particles

**DOI:** 10.3390/s17040929

**Published:** 2017-04-22

**Authors:** Chao Zhang, Dingqu Wang, Rong Zhu, Wenming Yang, Peng Jiang

**Affiliations:** 1 State Key Laboratory of Precision Measurement Technology and Instrument, Department of Precision Instrument, Tsinghua University, Beijing 100084, China; thzhangchao2014@163.com (C.Z.); jiang-p13@mails.tsinghua.edu.cn (P.J.); 2 Institute of Nuclear and New Energy Technology, Tsinghua University, Beijing 100084, China; wangdq@tsinghua.edu.cn; 3 School of Mechanical Engineering, University of Science and Technology Beijing, Beijing 100083, China; wmyang@ustb.edu.cn

**Keywords:** aerosol sensor, polydisperse ultrafine particle, size distribution, electrical detection, data fusion algorithm

## Abstract

Counting and sizing of polydisperse airborne nanoparticles have attracted most attentions owing to increasing widespread presence of airborne engineered nanoparticles or ultrafine particles. Here we report a miniature aerosol sensor to detect particle size distribution of polydisperse ultrafine particles based on ion diffusion charging and electrical detection. The aerosol sensor comprises a couple of planar electrodes printed on two circuit boards assembled in parallel, where charging, precipitation and measurement sections are integrated into one chip, which can detect aerosol particle size in of 30–500 nm, number concentration in range of 5 × 10^2^–5 × 10^7^ /cm^3^. The average relative errors of the measured aerosol number concentration and the particle size are estimated to be 12.2% and 13.5% respectively. A novel measurement scheme is proposed to actualize a real-time detection of polydisperse particles by successively modulating the measurement voltage and deducing the particle size distribution through a smart data fusion algorithm. The effectiveness of the aerosol sensor is experimentally demonstrated via measurements of polystyrene latex (PSL) aerosol and nucleic acid aerosol, as well as sodium chloride aerosol particles.

## 1. Introduction

Industrial environment, indoor and atmospheric contains airborne ultrafine particles and engineered nanoparticles [1,2], which pose a recognized health threat to hundreds of millions of people throughout the world [3,4,5]. In recent years, it is essential to have quantitative information on particle exposure levels for epidemiological and toxicological studies [6], searching for the relationship between human pathology and aerosol particle size distribution. A short review of human respiratory system illustrated a complexity relationship between human respiratory organs and airborne particles inhalation [7,8]. The researches indicate that the particle size smaller than about 10 µm which are accounted as PM_10_ (particulate matter with an aerodynamic diameter ≤10 µm) can enter nasal cavity, and the particle size smaller than about 2.5 µm which are accounted as PM_2.5_ can enter lungs [9,10], which become particularly pronounced in susceptible groups of the population such as infants, elderly and patients with chronic obstructive pulmonary disease (COPD). When the particle size further decreases to ultrafine particles or nanoparticles, the particles can enter alveolar area and even human blood circulation system [11,12], which arouse a higher risk to human health.

Size, number concentration [13] and surface area concentration [14] are the most fundamental parameters describing aerosol particles. A total particle population in which all the aerosol particles have the same size are said to be monodispersity, while aerosol particles that have a larger range in size are said to be polydispersity. In fact, the real particle size, either monodisperse or polydisperse aerosol particles, consists of a particle size distribution over a certain range. Particle size distribution represents an essential parameter for the evaluation of particle exposure/dose in each resided environment [15,16,17]. The way of quantitatively describing particle size distribution is categorized into number-averaged particle size, histograms of the number of particles in successive size intervals, and continuous distribution when the widths of the histograms approach zero and the histograms become a smooth continuous curve [2].

Electrical sensing for detection and counting of aerosol particles have been studied for decades [18]. Scanning mobility particle sizer (SMPS) or Fast mobility particle sizer (FMPS) serving as “gold-standard” instruments [19] are widely used in measurements of atmospheric, industrial environment and laboratory aerosol science researches. However, these instruments are not practicable for universal uses due to their big volume, heavy weight and high cost [20,21,22,23]. Some handheld instruments based on diffusion charging principle whereby the average charge on particles corresponds roughly to their diameters in a certain size range have been reported, such as Nanomonitor [24,25] and Discmini [26] devices. The Nanomonitor consists of three sections, i.e., charging, precipitation and sensing, which can measure the number concentration of ~10^6^ /cm^3^ and the averaged particle size between 10–300 nm. The Discmini [27] contains a diffusion stage and a filter stage, which can measure averaged particle sizes between 15–400 nm and number concentrations from 7 × 10^2^ to 8.4 × 10^5^ /cm^3^. We previously reported a micro aerosol sensor based on diffusion charging for detecting ultrafine particles in number concentration from 3 × 10^2^ to 2.5 × 10^4^ /cm^3^ and particle size from 50 to 253 nm [28]. However, these handheld instruments can only measure monodisperse particles or number-averaged particle size of polydisperse aerosol particles. Recently, portable particle size distribution analyzers, TSI NanoScan scanning mobility particle sizer (TSI NanoScan SMPS 3910, St Pual, MN, USA) [21,29] and Kanomax Portable Aerosol Mobility Spectrometer (Kanomax PAMS 3300, Osaka, Japan) [30], were reported. Both instruments apply the SMPS measuring scheme, utilizing a corona charger instead of a radioactive neutralizer, and integrating a differential mobility analyzer (DMA) and a condensation particle counter (CPC), the complexity of which hinders its further miniaturization and thus limits its wide applications.

In this work, we propose a miniature aerosol sensor based on diffusion charging principle, which enables detecting polydisperse airborne ultrafine particle size distribution by using a simple sensor structure with high-integration. The core component of the sensor is an aerosol sensing chip consisting of two parallel electrode plates, where three essential sections of the diffusion charging, precipitation and measurement are integrated into one chip with a size of 98 × 38 × 25 mm^3^. A novel measurement scheme by successively modulating the measurement voltages to trap particles with different sizes and deducing polydisperse particle size distribution through a data fusion algorithm is proposed. The aerosol sensor enables detecting aerosol particle size in range of 30–500 nm, number concentration in range of 5 × 10^2^–5 × 10^7^ /cm^3^. The sensor adopts a monolithic integration design which is compatible with micromachining, and thus enables further miniaturized. Compared with existing particle size analyzers, the proposed aerosol sensor is simple, miniature and low-cost, which has promising prospects in wide applications of personal environment monitoring and ultrafine aerosol particle detections for pollution monitoring and prevention.

## 2. System for Miniature Aerosol Sensor

### 2.1. Working Principle of the Aerosol Sensor

The miniature aerosol sensor is based on the fact that the average charge $\bar{q}$ per particle and the particle size *d_p_* have a certain degree of exponential relationship [31], which can be expressed as follow:

(1) $$\bar{q}(d_{p})=c\cdot {d_{p}}^{x}$$

where *c* is a constant determined through sensor calibration, *x* is a coefficient determined by the value of *N_i_*^.^*t_r_* (Fuchs theory) [2]. 

**Figure 1:**
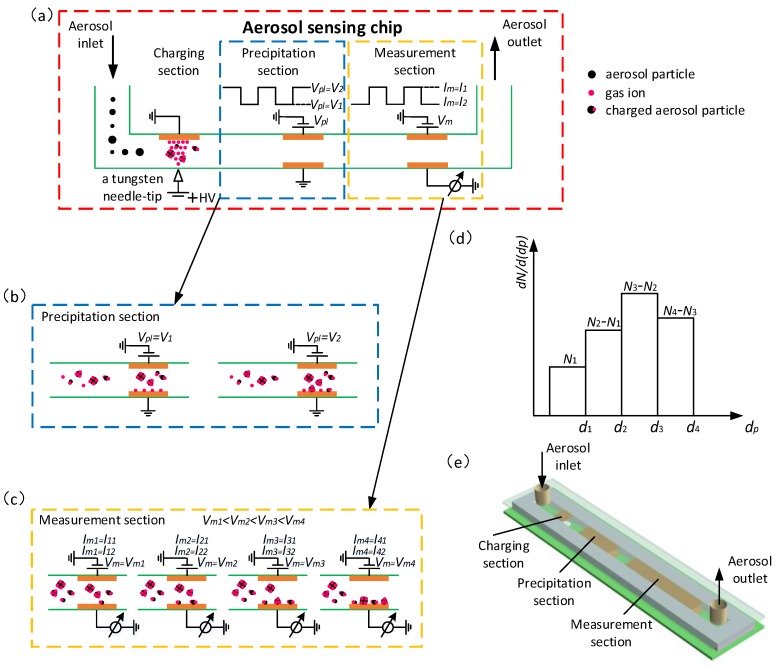
Working principle of aerosol sensor. (**a**) Schematic overview of the aerosol sensing chip with ion diffusion charging; (**b**) The motion behavior of the charged aerosol particles and gas ions in precipitation section; (**c**) The motion behavior of the charged aerosol particles in measurement section; (**d**) Histogram of particle number concentration versus particle size; (**e**) The structural diagram of aerosol sensing chip.

The working principle and the configuration of the aerosol sensor are shown in Figure 1. The aerosol sensing chip is a core component, which is composed of a micro channel (88 × 5 × 0.4 mm^3^) and a couple of planar electrodes printed on two circuit boards assembled in parallel, where three essential sections of charging, precipitation and measurement are integrated into one chip as shown in Figure 1a,e. The aerosol particle flow (0.4 L/min) is imported into the inlet of the flow channel. The aerosol particles are charged via ion diffusion charging in the charging section by a positive corona discharge generated between a tungsten needle-tip electrode and a planar electrode. The gas ions are ionized locally by the needle-tip electrode which is imposed sufficiently high positive voltage. The positive gas ions move from the needle-tip electrode to the opposite planar electrode forming a positive ion cloud. The aerosol particles are charged when they pass through the gas ion cloud via diffusion charging as shown in Figure 1a. 

After charging, the charged particles and excess gas ions enter into the precipitation section wherein they are subjected to an electric field modulated by a square voltage of *V*_1_ (low level) and *V*_2_ (high level) applied onto the two opposite planar precipitating electrodes with a frequency of 0.1 Hz as shown in Figure 1b. At the low level of *V*_1_, all excess gas ions are deposited onto the precipitating electrode, while almost all of the charged aerosol particles pass through the precipitation section and enter into the measurement section as shown in Figure 1b (left). At the high level of *V*_2_, all excess gas ions and a part of the charged aerosol particles are deposited onto the precipitating electrode, while the rest of charged aerosol particles pass through the precipitation section and enter into the measurement section as shown in Figure 1b (right). 

The charged aerosol particles entering into the measurement section are further controlled by a measurement voltage *V_m_* so that a part of charged particles are trapped onto the measuring electrodes and thereby export two total currents *I*_1_ and *I*_2_ in the measurement section corresponding to the low and high levels of the precipitation voltage *V*_1_ and *V*_2_ respectively. The measurement voltage *V_m_* is adjusted successively to different level (*V_m_*_1_ < *V_m_*_2_ < *V_m_*_3_ < *V_m_*_4_) to control the polydisperse aerosol particles trapped size-dependently onto the measuring electrodes, therefore a sequence of *I*_1_ (*I*_11_, *I*_21_, *I*_31_, *I*_41_) and *I*_2_ (*I*_12_, *I*_22_, *I*_32_, *I*_42_) are exported, which correspond to the modulated measurement voltage *V_m_* (*V_m_*_1_ < *V_m_*_2_ < *V_m_*_3_ < *V_m_*_4_) as shown in Figure 1c. In general, with the increase of the measurement voltage, more aerosol particles with a wider size range are trapped onto the measuring electrodes. The top measurement voltage *V_m_*_4_ drives all charged particles trapped onto the measuring electrodes. The exported sequence currents of *I*_1_ (*I*_11_, *I*_21_, *I*_31_, *I*_41_) and *I*_2_ (*I*_12_, *I*_22_, *I*_32_, *I*_42_) can obtained number concentration of *N* (*N*_1_, *N*_2_, *N*_3_, *N*_4_) and average particle size *d_p,av_*(*d_p,av_*_1_, *d_p,av_*_2_, *d_p,av_*_3_, *d_p,av_*_4_) by [28]

(2) $$N=S_{N}(I_{1}-I_{2})$$

(3) $$d_{p,av}=S_{d}\frac{I_{1}}{I_{1}-I_{2}}$$

where *S_N_* and *S_d_* are constant value. *N_i_* and *d_p,avi_* refer to total number concentration and average particle size in the aerosol particle size interval from 0 to *d_i_*, respectively.

Figure 1d shows the histograms of the number of particles in successive size intervals. According to the definition of the average particle size, *d_p,av_*_1_ and *d_p,av_*_2_ can be expressed as follows:

(4) $$d_{p,av1}=\frac{\int_{0}^{d_{1}}d_{p}dN(d_{p})}{N_{1}}$$

(5) $$d_{p,av2}=\frac{\int_{0}^{d_{2}}d_{p}dN(d_{p})}{N_{2}}=\frac{N_{1}d_{p,av1}}{N_{2}}+\frac{\int_{d_{1}}^{d_{2}}d_{p}dN(d_{p})}{N_{2}}$$

Based on (4) and (5), the average particle size in the particle size interval from *d*_1_ to *d*_2_ can be calculated as follows:

(6) $$d_{p,av(d_{1}-d_{2})}=\frac{N_{2}d_{p,av2}-N_{1}d_{p,av1}}{N_{2}-N_{1}}$$

Combining (6) with (2), the average particle size from *d*_1_ to *d*_2_ can be obtained:

(7) $$d_{p,av(d_{1}-d_{2})}=C\frac{I_{21}-I_{11}}{\left(I_{21}-I_{22})-(I_{11}-I_{12}\right)}$$

where *C* is calibration factor. Similarly, the average particle size *d_p,av___interval_* (*d_p,av__(0_*_-*d*_1_)_, *d_p,av__(d_*__1_-*d*_2_)_, *d_p,av__(d_*__2_-*d*_3_)_, *d_p,av__(d_*__3_-*d*_4_)_) for each size interval can be figured out by using the same method. In conclusion, the sequences of measurement currents *I*_1_ (*I*_11_, *I*_21_, *I*_31_, *I*_41_) and *I*_2_ (*I*_12_, *I*_22_, *I*_32_, *I*_42_) modulated by difference measurement voltages are directly related to the polydisperse particle size distribution expressed by the histograms of the number of particles in successive size intervals.

### 2.2. System for the Aerosol Sensor

**Figure 2:**
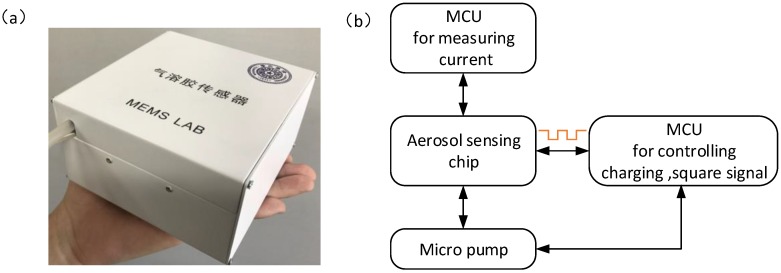
System for aerosol sensor. (**a**) A prototype of a packaged aerosol sensor system, including an aerosol sensing chip, a homemade signal conditioning circuit, and a micro pump assembled in a case; (**b**) The signal flow of the aerosol sensor system.

Figure 2 shows the system for the miniature aerosol sensor. The prototype of the aerosol sensor system is shown in Figure 2a. The system consists of an aerosol sensing chip, a homemade miniaturized control circuit board and a micro pump (Model T5-1LI, Parker, Cleveland, OH, USA), all of which are assembled in a case with 140 × 110 × 60 mm^3^. Figure 2b shows the signal flow of the aerosol system. The control circuit contains two micro controller units (MCUs), which are used to control corona discharge, generate square voltages and measure the output currents of the aerosol sensor. A tailor-designed conditioning circuit for measuring femto-ampere current level is also integrated in the system. The background noise of the developed aerosol sensor is in the low femto-ampere region with a standard deviation of 6.2 fA and a maximum deviation of 12.3 fA. While the measurement signal of the sensor is around pico-ampere, which is much larger than the noise and the detection limit of the measurement circuit.

### 2.3. Data Fusion Algorithm for Identifying Polydisperse Particle Size Distribution 

Theoretically, the output currents of the aerosol sensor directly respond to the polydisperse particle number concentrations in different size ranges as described in Section 2.1. However, due to complex and unideal motion behaviors of particles in the sensor channel, the relationship model *N_dp_* = *f* [*I*_1_, *I*_2_] between the sensor outputs *I*_1_ = (*I*_11_, *I*_21_, *I*_31_, *I*_41_), *I*_2_ = (*I*_12_, *I*_22_, *I*_32_, *I*_42_) and the polydisperse particle size distribution denoted as *N_dp_* = (*N_dp_* (0–50), *N_dp_* (50–100), *N_dp_* (100–150), *N_dp_* (150–200), *N_dp_* (200–250), *N_dp_* (250–300)) is a complex multivariate and nonlinear function. The variable *N_dp_* (*d*_1_–*d*_2_) refers to the total number concentration in the particle size interval from *d*_1_ to *d*_2_ nanometers. An approximation of *f* plays a crucial role in detection of polydisperse particle size distribution.

**Figure 3:**
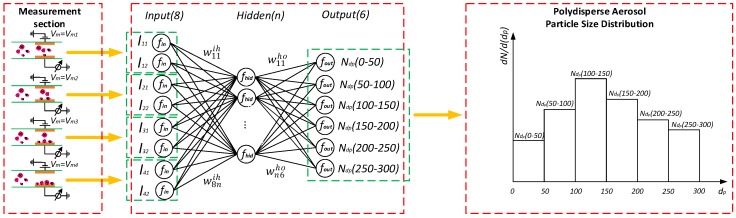
Structure of the neural network modeling the relationship between the aerosol sensor outputs and the polydisperse aerosol particle size distribution.

To solve the problem, we propose to adopt model identification method using a 3-layer back propagation (BP) neural network shown in Figure 3 to develop *f*, considering that it has been theoretically proved three layers of neural network could solve arbitrarily complicated nonlinear mapping problems. The network with 10 neurons in the hidden layer is used for the aerosol sensor, where the number of hidden neurons is determined through experimental trial from a small number up to the value when the decrease of the sum-squared network error became steady. In Figure 3, normalization function *f_in_* of the input layer, tan-sigmoid function *f_hid_* of the hidden layer, pure-line function *f_out_* of the output layer, and transfer matrix *w^ih^* and w*^ho^* constitute the model structure of the function *f*. The parameters *w^ih^* and w*^ho^* of the neural network model are determined through a calibration using experimental sample data. The training algorithm for calibration is the standard BP learning algorithm [32] with a learning rate of 0.1 that dominates the increment of the transfer weight in each iteration step and a momentum parameter of 0.9 that adjusts the efficiency of convergence in training to find optimal network parameters that minimize the errors between the network outputs and actual values. In real aerosol particle measurement, the developed neural network model is used to deduce the polydisperse particle size distribution *N_dp_* from the sensor outputs *I*_1_ and *I*_2_.

## 3. Experimental Setup and Aerosol Generation

**Figure 4:**
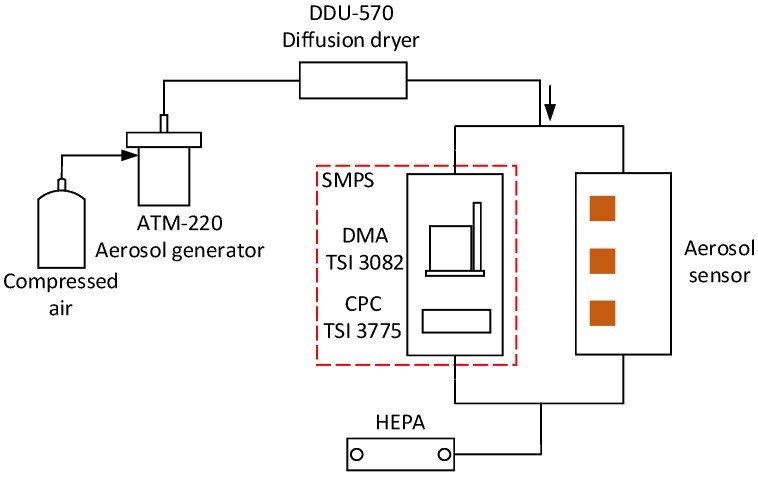
Experimental set up for testing the aerosol sensor.

The experimental setup for testing the developed aerosol sensor is depicted in Figure 4. The experimental system consists of an aerosol generator (ATM-220, TOPAS), diffusion dryer (DDU-570, TOPAS), SMPS (model 3938, TSI), high efficiency particulate air filter (HEPA, PALL 12144) and the developed aerosol sensor. The SMPS comprises a DMA (Model 3082, TSI) and a CPC (Model 3775, TSI). Aerosol particles including monodisperse aerosols and polydisperse aerosols are generated by the aerosol generator, and then the diffusion dryer absorbs excess water in the aerosols. The aerosol particles are measured by the SMPS to obtain the reference data, meanwhile the aerosol particles are measured by the developed aerosol sensor. A HEPA filter absorbs excess aerosol particles to prevent emissions into air.

Monodisperse polystyrene latex (PSL) aerosol is generated by using the aerosol generator and polystyrene latex liquid suspension (Thermo Scientific, Waltham, MA, USA). PSL nanospheres with the nominal diameters of 46, 69, 95, 209, 280 and 487 nm are used in the experiment. Monodisperse nucleic acid aerosol is generated by using the aerosol generator and coliform bacteria nucleic acids (DL 1000 and DL 5000, Thermo Scientific, Waltham, MA, USA). Through high performance liquid chromatography (HPLC) separation and purification, coliform bacteria nucleic acid sample with a certain single fragment length is prepared. Standard duplex coliform bacteria DNA with chain lengths of 100–700 base pairs (bp) corresponding to the fragment length of 34–238 nm are used in the experiment.

Polydisperse sodium chloride (NaCl) aerosols are generated by using the aerosol generator and NaCl solution. NaCl solutions with concentrations of 50 mg/L, 200 mg/L, 1 g/L, 10 g/L and 50 g/L are used to generate polydisperse aerosol particles with maximum size of 37, 58, 100, 215 and 368 nm (from instruction manual of ATM-220 aerosol generator), respectively.

## 4. Results and Discussion

### 4.1. Preliminary Experiments

A series of preliminary experiments are conducted before aerosol measurement. The working parameters of the developed aerosol sensor, including corona discharge voltage, square voltages applied on the precipitating electrodes, and measurement voltage applied on the measuring electrodes, are optimally determined. Firstly, the corona discharge voltage of the aerosol sensor is determined by gradually increasing the voltage on the charging section until the corona discharge occurs and self-sustains. Then the discharge voltage is maintained. Through this experiment procedure, the discharge voltage is finally set as 1400 V for the sensor. 

And then the top measurement voltage *V_m_*_4_ is determined by increasing the measurement voltage until the output current achieves saturation while the precipitating electrodes are floated, which ensures all charged aerosol particles trapped in the measurement section. The precipitation square voltages are determined by the way that the low voltage is used for trapping all excess gas ions except the charged aerosol particles onto the precipitating electrodes and the high voltage is for trapping all excess gas ions and the charged aerosol particles with minimal size to be detected onto the precipitating electrodes. For our developed aerosol sensor, the corona voltage is finally set as 1400 *V*, the top measurement voltage is set as 5 *V*, the low and high voltages of the precipitation square signal are set as 0.8 *V* and 3 *V* respectively. To detect monodisperse particles, a uniform measurement voltage *V_m_* = 5 *V* is used in the measurement section. To detect polydisperse particles, a sequence of measurement voltages *V_m_*_1_ = 2 *V*, *V_m_*_2_ = 3 V, *V_m_*_3_ = 4 V, *V_m_*_4_ = 5 V are used successively in the measurement section. 

### 4.2. Measurement Results and Discussion

**Figure 5:**
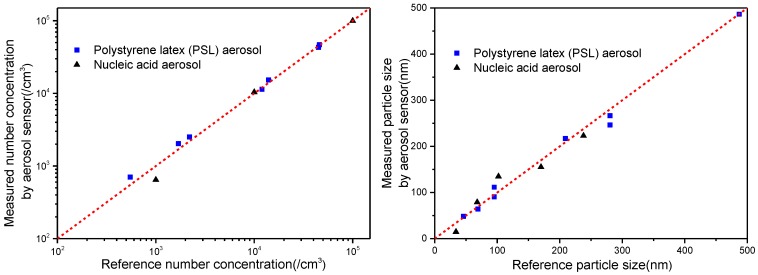
Comparison of measured particle number concentration (**left**) and particle size (**right**) with reference data for monodisperse aerosols.

To characterize the developed aerosol sensor, sorts of monodisperse ultrafine particles are detected using the sensor. Polystyrene latex (PSL) nanospheres custom-made with specific size are commonly used as standard nanoparticles. Therefore PSL aerosols with certain particle sizes are used in the experiment to test the developed aerosol sensor. The measured particle size and number concentration by the sensor are compared with the reference data by the SMPS as shown in Figure 5. Besides of the synthetic polymer particles, natural polymer particles (e.g., nucleic acid molecules) are also detected using the aerosol sensor. Nucleic acids are linear biopolymers (chains) of nucleotides, or large biomolecules, essential for all known forms of life. Different chain lengths of nucleic acids correspond to different effective sizes [33]. The comparison of the measured number concentration (left) and size (right) of the nucleic acid aerosols to the reference data are also shown in Figure 5. The results present satisfactory agreement between the measured results and the reference data for detecting monodisperse aerosols with the particle size in range of 30–500 nm and the number concentration in range of 5 × 10^2^–5 × 10^5^ /cm^3^. Taking all samples together, the average relative errors of the measured aerosol number concentration and the particle size from the reference data are estimated to be 12.2% and 13.5% respectively.

**Figure 6:**
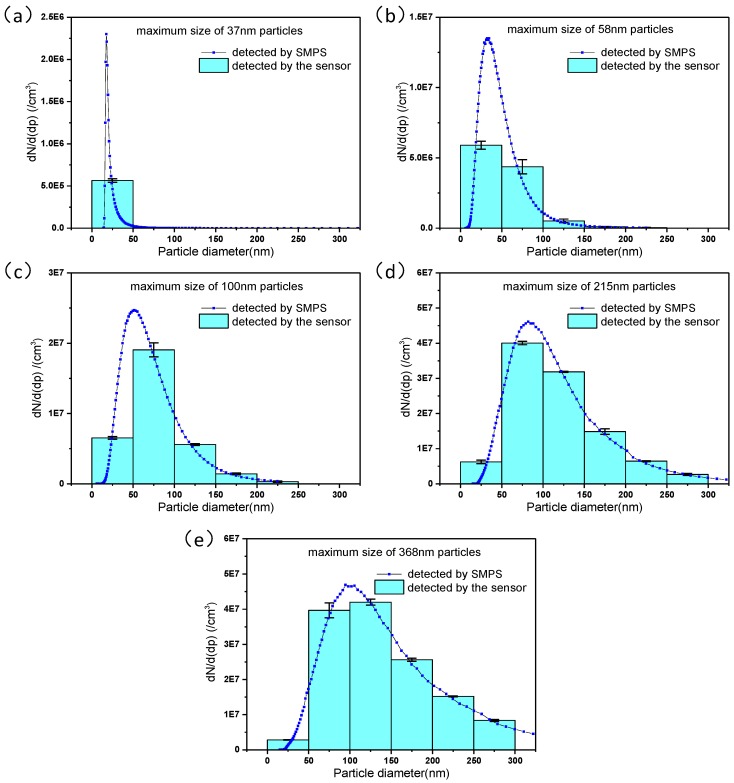
Comparison of NaCl particle size distribution measured by the aerosol sensor and the reference data for polydisperse NaCl aerosol. Error bars from three repeated measurement are included. Maximum sizes of polydisperse NaCl particles are (**a**) 37 nm, (**b**) 58 nm, (**c**) 100 nm, (**d**) 215 nm, and (**e**) 368 nm, respectively.

**Figure 7:**
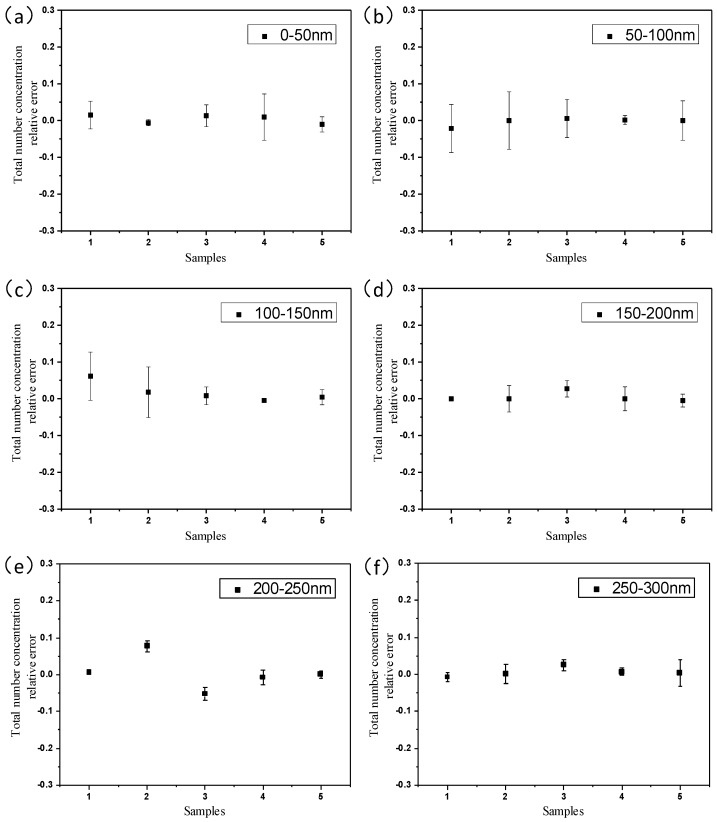
Relative errors of total number concentration in different aerosol particle size interval of (**a**) 0 to 50 nm, (**b**) 50 to 100 nm, (**c**) 100 to 150 nm, (**d**) 150 to 200 nm, (**e**) 200 to 250 nm, (**f**) 250 to 300 nm. Error bars from three repeated measurements are included.

To further verify the feasibility of the aerosol sensor for detecting polydisperse particle size distribution, the polydisperse sodium chloride (NaCl) aerosols with different particle size distributions generated by the aerosol generator are detected using the aerosol sensor, results of which are compared with the reference particle size distribution characterized by the SMPS. The polydisperse NaCl particles with difference size distributions are used in the experiment. The histograms of the number concentration of particles in successive size intervals that are deduced from the developed neural network by importing the sensor output currents are compared with the reference particle size distribution as shown in Figure 6, which demonstrates that the results measured by the developed aerosol sensor agree with the reference particle size distribution very well. The relative errors of the measurements are analyzed and shown in Figure 7, which indicates that the average relative errors of the total number concentration in the particle size intervals of 0–50 nm, 50–100 nm, 100–150 nm, 150–200 nm, 200–250 nm and 250–300 nm are 1.1%, 0.57%, 1.9%, 0.64%, 2.9% and 0.86% respectively.

## 5. Conclusions

In this paper, we propose a miniature aerosol sensor for detecting aerosol particle size in range of 30–500 nm, number concentration in range of 5 × 10^2^– 5 × 10^7^ /cm^3^ based on diffusion charging and electrical measurement. The sensor is simple and highly integrated by assembling two parallel circuit boards to form a micro channel, on which a couple of planar electrodes used for corona discharge, particle precipitation and particle measurement are integrated. A polydisperse airborne particle size distribution is detected by successively modulating the measurement voltage applied on the measuring electrodes to control the precipitation rate of the charged aerosol particles. A neural network based data fusion algorithm is utilized to deduce the particle size distribution by the time series measuring currents. The methodology of diffusion charging and electrical measurement incorporated with a smart data fusion algorithm allows simplification and miniaturization of a polydisperse aerosol sensor, and thus expands its application scope and lowers cost. Experiments on polystyrene latex aerosol, nucleic acid aerosol, and polydisperse sodium chloride particles validate the effectiveness of the aerosol sensor for measuring diverse ultrafine aerosol particles. In our future study, we will further conduct field measurement and comparison using the miniature aerosol sensor for practical applications, such as indoor and outdoor air monitoring. Furthermore, quantitative analysis of particle loss and charging will be carried out to improve the sensor.
